# Supplementary figures and images for: Psychologic stress and disease activity in patients with inflammatory bowel disease: A multicenter cross-sectional study
Source: PLoS One. 2020 May 26;15(5):e0233365. doi: 10.1371/journal.pone.0233365 (PMC7250441; doi:10.1371/journal.pone.0233365)

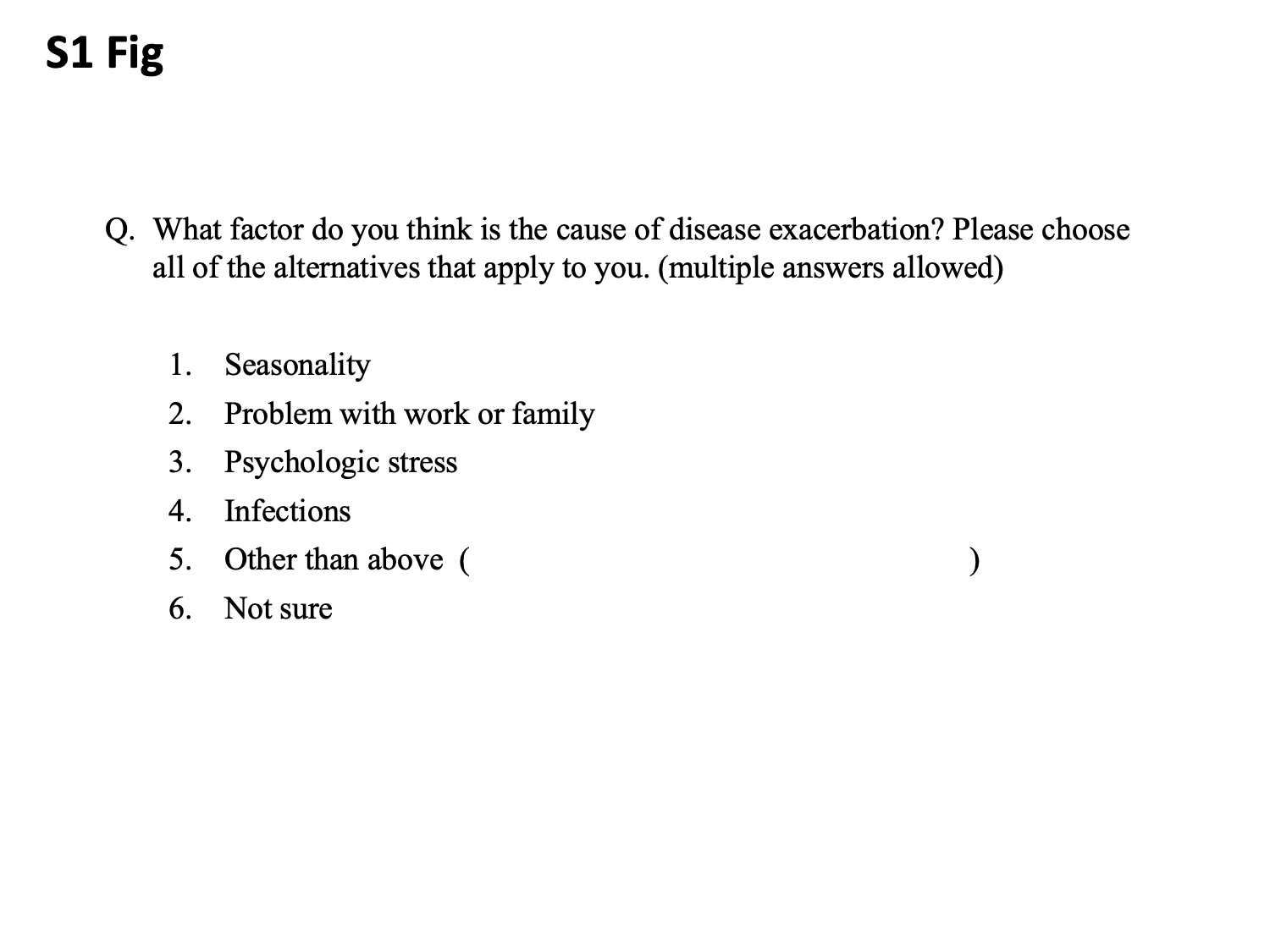

Supplement: S1 Fig — (TIFF) [file pone.0233365.s001.tiff]

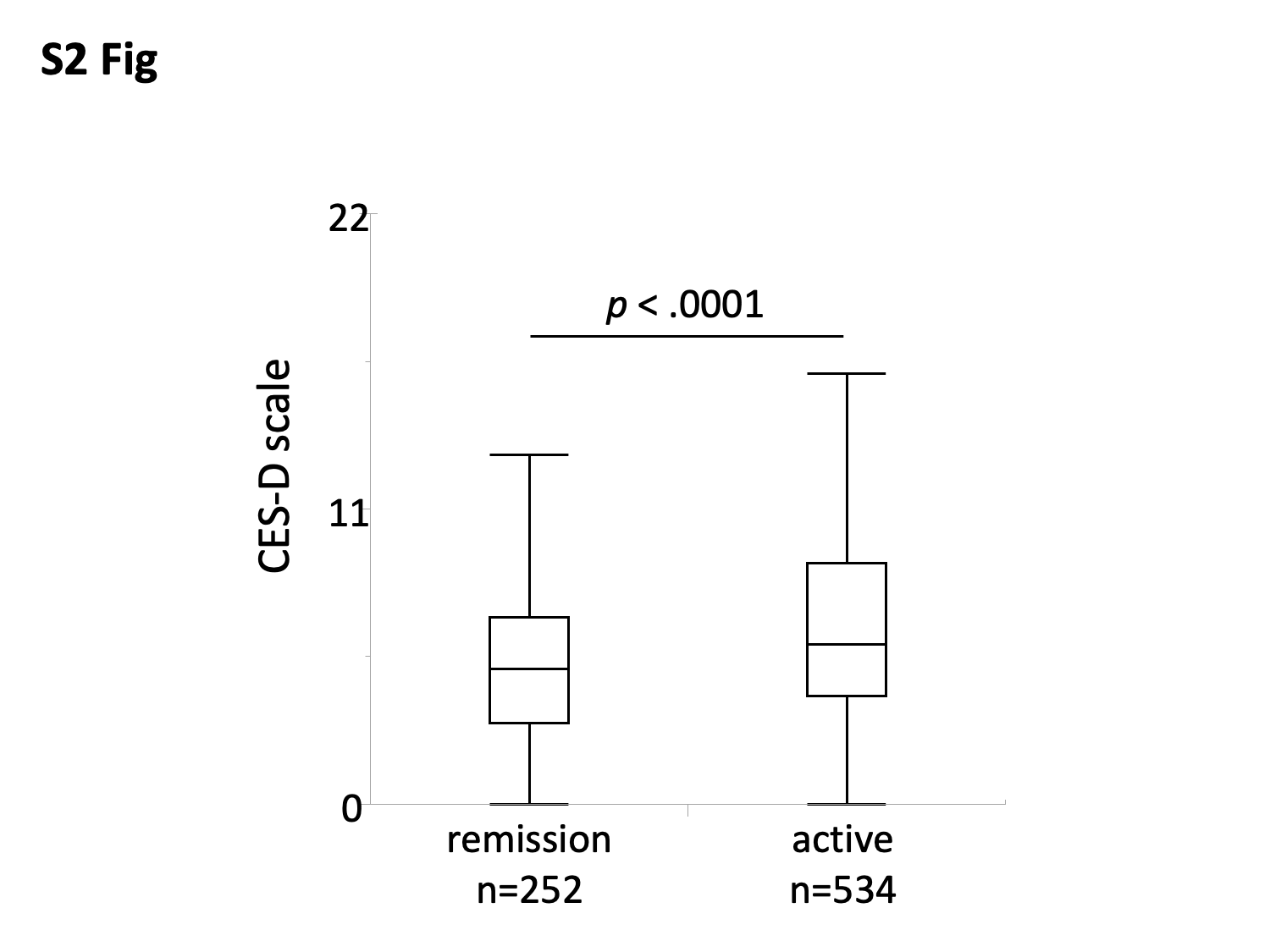

Supplement: S2 Fig — In all IBD patients, the CES-D scores were significantly higher for patients with active disease than for those in remission (median (IQR) = 6 (4–9) vs. 5 (3–7), p < .0001). (TIFF) [file pone.0233365.s002.tiff]

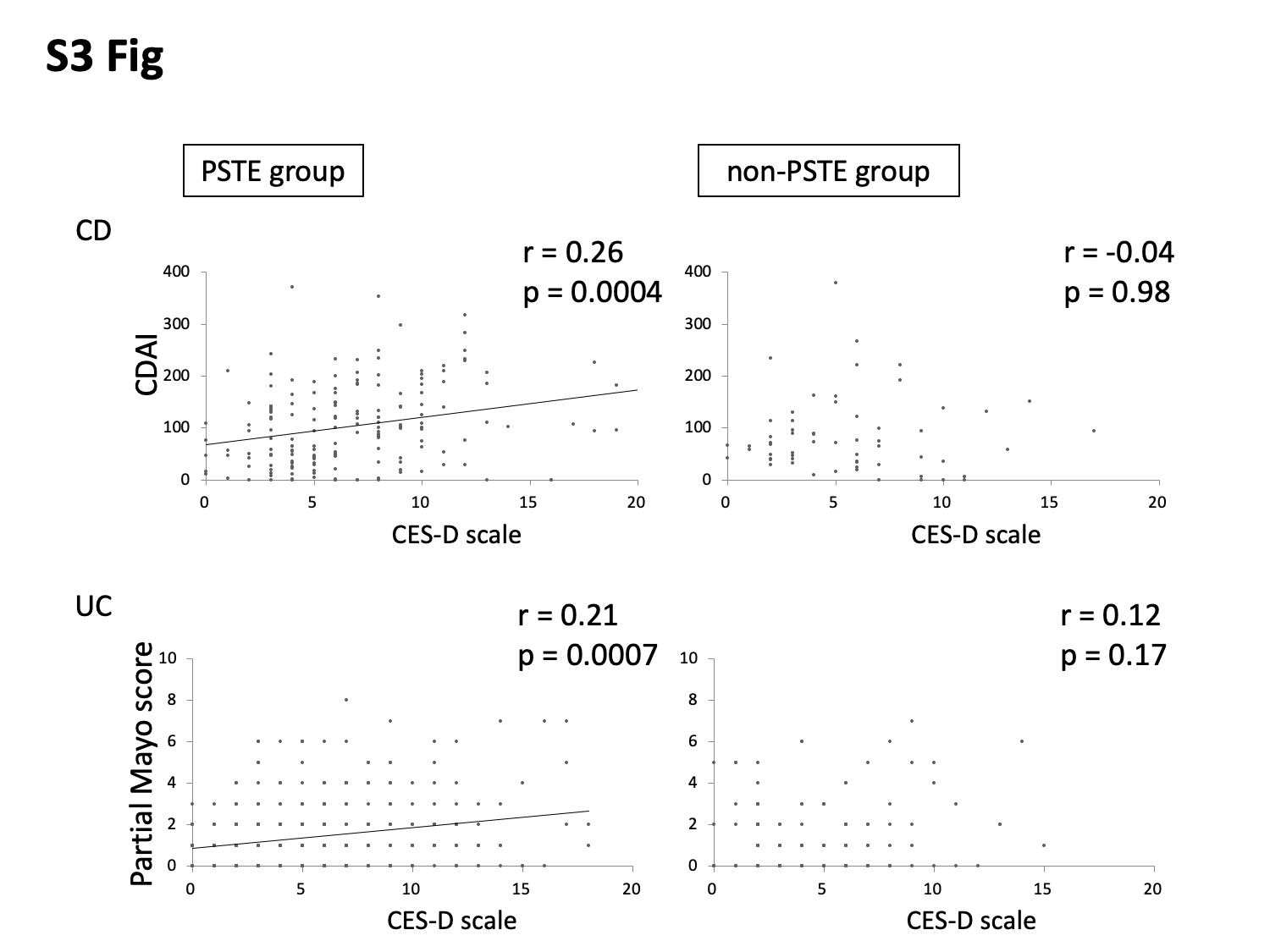

Supplement: S3 Fig — In the PSTE group, the CES-D scores correlated positively with disease activity (r = 0.26, p = 0.0004 in CD and r = 0.21, p = 0.0007 in UC). In patients with CD (n = 60) and UC (n = 133) in the non-PSTE group, the CES-D scores did not correlate with disease activity (r = -0.04, p = 0.98 in CD and r = 0.12, p = 0.17 in UC). (TIFF) [file pone.0233365.s003.tiff]

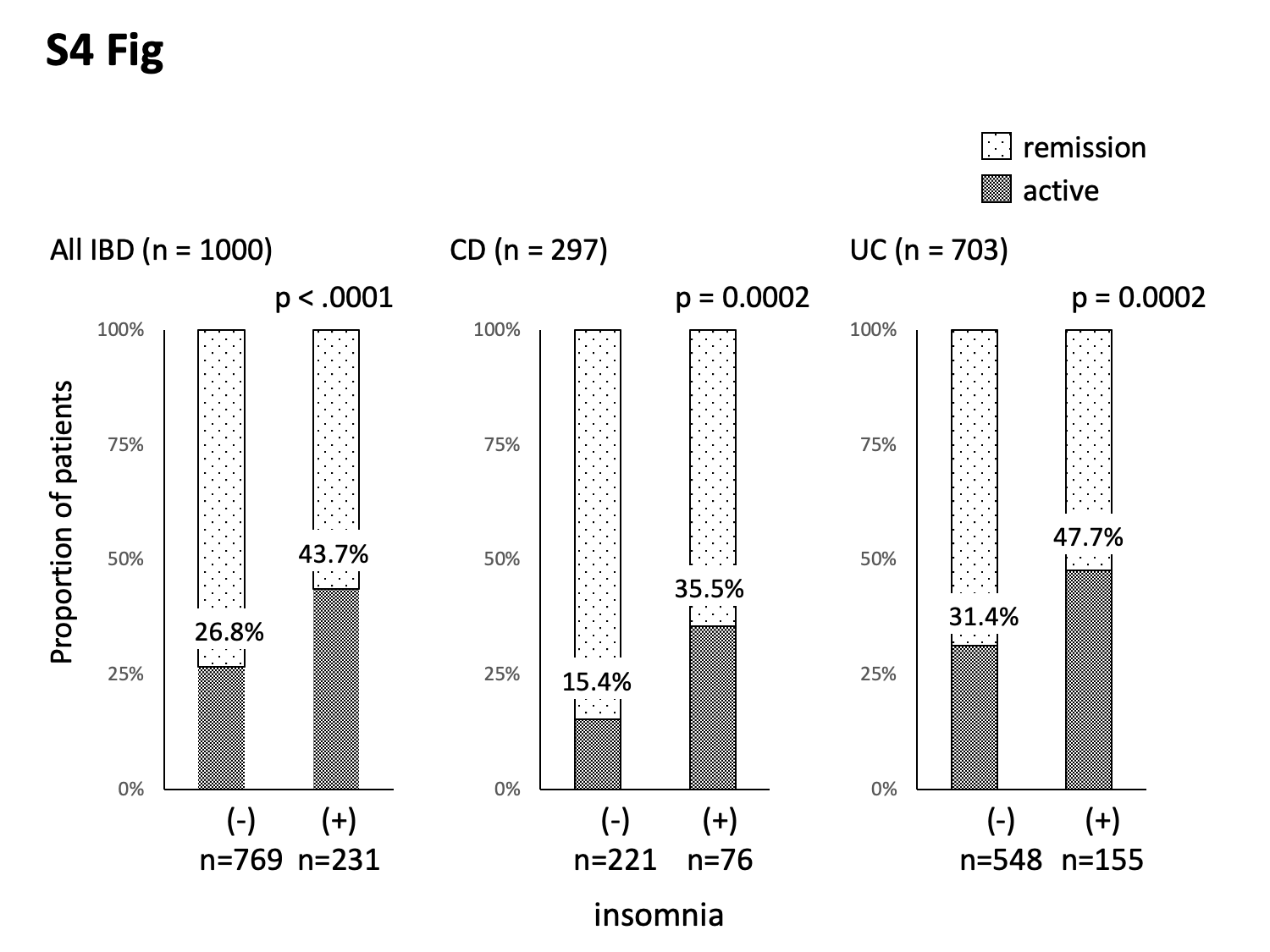

Supplement: S4 Fig — Among patients with CD and UC, the proportion of patients with active disease was significantly higher in the insomnia (+) group than in the insomnia (-) group. (TIFF) [file pone.0233365.s004.tiff]

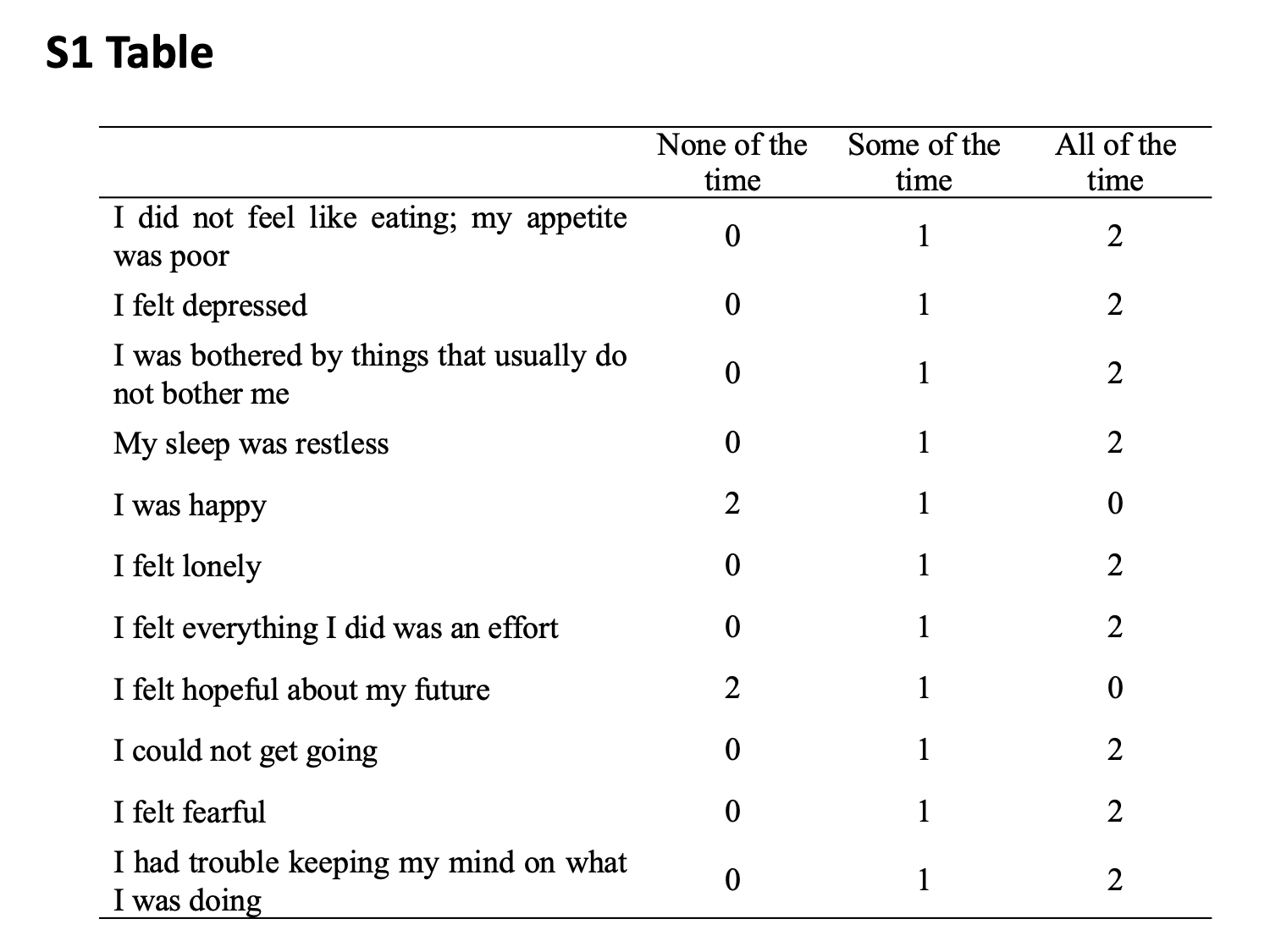

Supplement: S1 Table — (TIFF) [file pone.0233365.s005.tiff]
