# Supplementary material for: Psychologic stress and disease activity in patients with inflammatory bowel disease: A multicenter cross-sectional study
Source: PLoS One. 2020 May 26;15(5):e0233365. doi: 10.1371/journal.pone.0233365 (PMC7250441; doi:10.1371/journal.pone.0233365)
Supplement: Attachment — Submitted filename: Araki Stress Reponse to Reviewers v2.4.pdf [file pone.0233365.s006.pdf]

Response to Reviewer #1:

Thank you very much for reviewing our manuscript. We have revised the manuscript in response to all comments received.

1. The word, "vulnerability to psychological stress" may be improper because the authors did not measure the vulnerability such as lack of stress coping. I assume that "vulnerability to psychological stress" means that patients believed that psychological stress triggered their disease exacerbation in this study. This concept may be better to have a different name such as patients' belief.

<Answer> Thank you for this suggestion. As the reviewer pointed out, we intended to investigate patients who believed that psychologic stress triggered an exacerbation of their disease. In the revised manuscript, we changed the title and amended the related sentences explaining the concept of the patients' belief.

2. Instead of psychological stress, the authors measure depression (CES-D) and insomnia. The concept of psychological stress is different from depression and insomnia. The authors should clearly use the word such as depression and insomnia instead of psychological stress when they discuss depression and insomnia. In other words, the author should only use the word "psychological stress" when they discuss the results of questionnaire regarding factors related to disease exacerbation.

<Answer> To avoid misunderstanding, we used the phrase "psychologic stress" only when discussing the results of the questionnaire, and used the phrase "depressive state" when referring to the association between CES-D scores and disease activity.

3. I believe that the most important information of this article may be that psychological stress was the most common factor for a trigger of their disease

exacerbation. The authors should write the exact sentences to shown how they asked this question and what the choices were.

<Answer> The exact sentences of the question are now provided on **lines 143-147** in the revised manuscript. And according to the suggestion by the Journal Requirements, we have also included the original Japanese version as **S1 Fig** in Supporting Information.

4. The authors used the word group 1 and group 2 which were based on whether patients answered whether psychological stress triggered their disease exacerbation. The expression of group 1 or group 2 may not be easy to follow for readers because name of group numbers (group 1 and group 2) are confusing. Please rename group1 and group 2 for readers' understanding.

<Answer> We renamed group 1 and group 2 as the PSTE (psychologic stress-triggered exacerbation) group and the non-PSTE group. Thank you for this suggestion.

5. It seems that authors tried to show there is effect modification (i.e. interaction) between group 1 and group 2 regarding the association between CES-D or insomnia and disease activity. In the current analysis, the authors conducted only subgroup analyses based on group 1 and group2. Please show the p-value for interaction. Based on the p-value for interaction, the authors can discuss whether depression or insomnia are associated with disease activity in Group1 but not in Group 2.

<Answer> Figure 2 (new **Fig 3**), which shows the association between the CES-D scores and disease activity in each group, indicates a tendency toward an interaction with a p-value of 0.066. This result indicates a positive association between CES-D scores and disease activity only in the PSTE group and no association was observed in the non-PSTE group. As for Figure 5 (new **Fig 4**),

which shows the association between the clinical activity scores and insomnia in each group, the p-values for the group interactions were 0.61 for CD and 0.23 for UC, respectively. These data indicate a positive association between insomnia and disease activity, especially in the PSTE group. Although significant differences were not observed in the non-PSTE group, an association between insomnia and disease activity cannot be ruled out. Further studies are needed to clarify this point, and we are now conducting a prospective study. We amended our description of the results in **lines 195-196** and **246-249**, and added sentences to the Discussion (**lines 310-318**) in the revised manuscript.

6. The authors used CES-D as a continuous variable and showed the significant difference between remission and active patients in Group 1 in Figure 2. It may be better to use categorical variable such as depressive or not based on the cut-off written in the method section. If the authors use CES-D as a continuous variable, please discuss minimal clinically important difference (MCID) of CES-D.

<Answer> We re-analyzed the data using categorical variables with Fisher's exact test, and showed that a higher proportion of active patients were in a depressive state than in a non-depressive state in the PSTE group, but this difference was not observed in the non-PSTE group.

		Depressive state	Non-depressive state	p-value
Active disease, n(%)	PSTE group	97 (42.9)	96 (26.2)	<.0001
	Non-PSTE group	18 (34.0)	41 (29.3)	0.6001

As shown in Supplementary Figure 4 (new **S3 Fig**), however, the CES-D score correlated significantly with the clinical activity indices in the PSTE group, but not in the non-PSTE group, although both scores were used as continuous variables. Therefore, we would like to present the data as continuous variables.

There is no defined MCID for the CES-D score, but, according to the reviewer's suggestion, we added the median values and interquartile range (IQR) in the figure legend of new **Fig 3** (former Figure 2) in the revised manuscript.

7. Instead of table 1 and table 2, it may be better to show only patient characteristics in all patients, Group 1, and Group 2 in the same table without any statistical testing. In other words, the authors may delete table 1 and table 2, and they create a new table showing the patient characteristics.

<Answer> In response to this comment, we now provide the patient characteristics in the new Supplementary Table 1, which combines the previous Table 1 and 2, and relabeled the previous Supplementary Table 1 as a new **Table 1**. We understand that showing all the factors in univariate analyses is not always necessary, but we also consider that the multivariate analyses shown in the old Table 1 and 2 are very important data. We therefore revised these tables by showing only the factors for which multivariate analyses were performed (new **Table 2 and 3**, respectively).

8. I believe that the supplementary figure 1 is important because it shows patients' selection. Please transfer the supplementary figure 1 to figure 1 in the manuscript.

<Answer> We modified and moved Supplementary Figure 1 to **Fig 1**, as suggested by the reviewer in comment No.10.

9. There may be too many figures and supplementary figures. Please decrease the number of figures only focus on exacerbation factors and the subgroup analysis of Group 1/2 regarding depression and insomnia because this may be a main research question of this study. If this is not, the authors should show what is their main research question.

<Answer> During the review process, we decided that analyses of the detailed insomnia symptoms in the insomnia (+) and insomnia (-) groups did not need to be shown in the present form. We therefore deleted the previous Figure 4, Supplementary Figure 6, and related descriptions in the manuscript were modified. We also deleted previous Supplementary Figures 2 and 5 in which the proportions of patients are shown, and the related descriptions in the manuscript were amended.

10. As shown in the supplementary figure 1, the authors selected the study population in two ways. If the authors decrease the number of figures and analyses, it may be possible to select patients in one process.

<Answer> As we performed the depression and insomnia analyses similarly, we amended the new **Fig 1** (former Supplementary Figure 1) in which the study is shown as one process.

11. In the overall analyses, the authors showed only p-values in figures. Please show the point estimates such as mean difference and 95% intervals. This is important to discuss whether the difference is clinically important or not. This issue is related to MCID as I commented in No 6.

<Answer> For analyses with continuous variables by which the point estimates can be shown such as in Figure 2 (new **Fig 3**), Figure 5 (new **Fig 4**), and Supplementary Figure 3 (new **S2 Fig**), we show median values with the IQR described in the figure legends. For analyses with categorical variables such as in Figure 3 (new **S4 Fig**), we show the number and ratio of patients in each group.

12. The authors should carefully interpret results in this study. Because this is the cross-sectional study, it is reasonable to consider the depression or insomnia is due to

disease activity rather than vice-versa. Please discuss the results from the viewpoint above. Moreover, the authors should explain how patients' belief (psychological stress triggered their disease exacerbation) affects the association of depression or insomnia with disease activity. In other word, please explain the mechanism of this effect modification if the authors find the significant interaction (please see the comments No 5).

<Answer> As described in the limitation section, we understand that this study design does not clarify whether a depressive state or insomnia are a cause or consequence of the exacerbated symptoms, and would like to emphasize that when patients are divided into PSTE and non-PSTE groups, different associations between the disease activity and depressive state/insomnia are observed in each group. The findings of an effect modification differed between depressive state and insomnia, indicating that a patient's belief in psychologic stress-triggered disease exacerbation is strongly connected to the association between the depressive state and disease activity, although an association between insomnia and disease activity cannot be ruled out. A depressive state might be more strongly involved than insomnia in disease activity in patients recognizing psychologic stress-triggered disease exacerbation, but in the present study design, a direct comparison cannot be performed and a prospective study targeting this issue is needed. Thank you for the important suggestion and we have now added sentences addressing this point in **lines 319-327** of the revised manuscript.

13. Overall, there may be better words and sentences to show what the authors want to discuss in this manuscript. Please ask English editing to native and academic English editors.

<Answer> The manuscript was edited by professional native-English speaking science editors from SciTechEdit International, LLC.

1. It is better to show the study design in detail in the title. This is a multicenter cross-sectional survey or multicenter cross-sectional study.

<Answer> We changed the title to indicate that this was a multicenter cross-sectional study.

2. In the introduction, the authors do not need to discuss seasonality because this is different from the current study purpose.

<Answer> We deleted the sentences describing seasonality in the Introduction.

3. In the method section, please briefly mention the validity and reliability of CES-D and insomnia questionnaire based on previous questionnaire development study conducted by the developers.

<Answer> The CES-D scale was validated by Yokoyama E, et al. (Sleep. 2010. Reference No.21) As for the insomnia questionnaire, we used a simple version of the sleep questionnaire based on the validated Insomnia Severity Index (ISI) (Bastien CH, et al. Sleep Med. 2001, which has been added as Reference No. 23) to reduce recall bias. It is commonly given to patients to assess their present sleep conditions in clinical practice. We briefly described the questionnaire in **lines 164-166** of the revised manuscript.

4. In the introduction and discussion, the author should summarize previous studies evaluating patients' belief of disease exacerbation triggers and discuss the discrepancy between the results of current study and those of previous studies with possible reasons.

<Answer> Patients' beliefs about some environmental factors affecting IBD, such as diet (Limdi JK, et al. Inflamm Bowel Dis. 2016, which has been added as Reference No. 15) and smoking (Saadoune N, et al. Eur J Gastroenterol Hepatol. 2015, which has been added as Reference No. 16) have been reported, but no studies have focused on patients' beliefs regarding psychologic stress and disease activity. The present data provide new insight that a depressive state is one of the factors associated with disease activity in a subgroup of IBD patients who believed that psychologic stress triggered an exacerbation of their disease. We now describe this in **lines 91-93** in the Introduction and **lines 282-285** in the Discussion of the revised manuscript.

5. In the introduction and discussion, the author should summarize previous studies evaluating depression or insomnia and disease activity, and please discuss the discrepancy between the results of current study and those of previous studies with possible reasons.

<Answer> As described in the Introduction, there are conflicting reports regarding whether psychologic state and insomnia are associated with disease activity. In the present study, we found that depressive state is a factor associated with disease activity in a subgroup of IBD patients who believed that psychologic stress triggered an exacerbation of their disease. Previous conflicting reports might be due to the lack of patient stratification based on patients' belief. We now discuss this in **lines 285-287** of the revised manuscript.

Response to Reviewer #2:

Thank you very much for reviewing our manuscript. We have revised the manuscript in response to all comments received.

1. the discussion section should be altered to more accurately reflect the cross-sectional nature of the data. As an example, the statement " .. and the results suggest that psychological stress can be an initiator of disease" (line 281) is simply not appropriate. Similarly, the statement "our present data also suggest that high vulnerability to psychologic stress is associated with disease flare in patients with insomnia" (line 273) is not supported by the data.

<Answer> Thank you for the suggestion. We agree and have deleted these statements to avoid misunderstanding.

2. Finally, their conclusion "In conclusion, our data revealed that psychologic stress and insomnia affect disease activity in IBD patients, especially..." (line 292) is wrong, since it is just as likely that disease activity affects psychological stress and insomnia.

<Answer> As we described in the limitation section, in the present study, we could not clarify whether psychologic stress and insomnia affect disease activity or vice versa. Therefore, we changed the statement to "worsened mental state correlates with disease activity in IBD patients, especially..." in **lines 342-343** of the revised manuscript, and deleted the word "insomnia" because of negative results regarding an effect modification between the PSTE and non-PSTE groups. Thank you for your suggestion.

Response to Journal Requirements:

When submitting your revision, we need you to address these additional requirements.

1. Please ensure that your manuscript meets PLOS ONE's style requirements, including those for file naming. The PLOS ONE style templates can be found at http://www.journals.plos.org/plosone/s/file?id=wjVg/PLOSOne_formatting_sample_main_body.pdf and http://www.journals.plos.org/plosone/s/file?id=ba62/PLOSOne_formatting_sample_title_authors_affiliations.pdf

<Answer> We amended the manuscript so as to meet PLOS ONE's style requirements.

2. Please provide additional details regarding participant consent. In the ethics statement in the Methods and online submission information, please ensure that you have specified (1) whether consent was informed and (2) what type you obtained (for instance, written or verbal, and if verbal, how it was documented and witnessed). If your study included minors, state whether you obtained consent from parents or guardians. If the need for consent was waived by the ethics committee, please include this information.

<Answer> The written informed consent was waived by the ethics committees by giving the participants the opportunity to opt out. We described this on **lines 125-126** of the revised manuscript.

3. Please include additional information regarding the questionnaire regarding potential factors of disease exacerbation used in the study and ensure that you have provided sufficient details that others could replicate the analyses. For instance, if you developed this questionnaire as part of this study and it is not under a copyright more restrictive than CC-BY, please include a copy, in both the original language and English, as Supporting Information.

<Answer> According to the suggestion by the Reviewer #1, we described the detailed information of the questionnaire in English on the Methods of the revised manuscript. We have also included the original Japanese version as **S1 Fig** in Supporting Information.

4. Thank you for stating the following in the Acknowledgments Section of your manuscript:
'This work was supported by a Grant-in-Aid from the Japan Society for the Promotion of Science (Grant No. 26460969).'
We note that you have provided funding information that is not currently declared in your Funding Statement. However, funding information should not appear in the Acknowledgments section or other areas of your manuscript. We will only publish funding information present in the Funding Statement section of the online submission form.
Please remove any funding-related text from the manuscript and let us know how you would like to update your Funding Statement. Currently, your Funding Statement reads as follows:
'The authors received no specific funding for this work.'

<Answer> We have removed the funding-related text from the manuscript and updated the Funding Statement section in the online submission form.

5. Thank you for stating the following in the Competing Interests section:
'The authors have declared that no competing interests exist.'
We note that one or more of the authors are employed by a commercial company: Kinshukai Infusion clinic

<Answer> Kinshukai Infusion clinic is not a commercial company but a medical corporation. The author has declared that no competing interests exist.

1. Please provide an amended Funding Statement declaring this commercial affiliation, as well as a statement regarding the Role of Funders in your study. If the funding organization did not play a role in the study design, data collection and analysis, decision to publish, or preparation of the manuscript and only provided financial support in the form of authors' salaries and/or research materials, please review your statements relating to the author contributions, and ensure you have specifically and accurately indicated the role(s) that these authors had in your study. You can update author roles in the Author Contributions section of the online submission form.
Please also include the following statement within your amended Funding Statement.
“The funder provided support in the form of salaries for authors [insert relevant initials], but did not have any additional role in the study design, data collection and analysis, decision to publish, or preparation of the manuscript. The specific roles of these authors are articulated in the ‘author contributions’ section.”
If your commercial affiliation did play a role in your study, please state and explain this role within your updated Funding Statement.

<Answer> This work was supported by a Grant-in-Aid from the Japan Society for the Promotion of Science (Grant No. 26460969). The funder did not have any additional role in the study design, data collection and analysis, decision to publish, or preparation of the manuscript. We have now updated the Funding Information of the online submission system and added above statements in **lines 359-362** in Author Contributions of the revised manuscript.

2. Please also provide an updated Competing Interests Statement declaring this commercial affiliation along with any other relevant declarations relating to employment, consultancy, patents, products in development, or marketed products, etc.
Within your Competing Interests Statement, please confirm that this commercial affiliation does not alter your adherence to all PLOS ONE policies on sharing data and materials by including the following statement: "This does not alter our adherence

to PLOS ONE policies on sharing data and materials." (as detailed online in our guide for authors http://journals.plos.org/plosone/s/competing-interests). If this adherence statement is not accurate and there are restrictions on sharing of data and/or materials, please state these. Please note that we cannot proceed with consideration of your article until this information has been declared.
Please include both an updated Funding Statement and Competing Interests Statement in your cover letter. We will change the online submission form on your behalf.
Please know it is PLOS ONE policy for corresponding authors to declare, on behalf of all authors, all potential competing interests for the purposes of
transparency. PLOS defines a competing interest as anything that interferes with, or could reasonably be perceived as interfering with, the full and objective presentation, peer review, editorial decision-making, or publication of research or non-research articles submitted to one of the journals. Competing interests can be financial or non-financial, professional, or personal. Competing interests can arise in relationship to an organization or another person. Please follow this link to our website for more details on competing interests: http://journals.plos.org/plosone/s/competing-interests

<Answer> We have read the journal's policy and declare that the authors have no competing interests which can alter our adherence to PLOS ONE policies on sharing data and materials. We have amended the Disclosure Statement on **lines 373-374** of the revised manuscript, and the cover letter.

6. Thank you for including your ethics statement: The ethics committees of all participating institutions approved the study protocol.
Please amend your current ethics statement to include the full name of the ethics committee/institutional review board(s) that approved your specific study.
Once you have amended this/these statement(s) in the Methods section of the manuscript, please add the same text to the "Ethics Statement" field of the submission form (via "Edit Submission").

For additional information about PLOS ONE ethical requirements for human subjects research, please refer to http://journals.plos.org/plosone/s/submission-guidelines#loc-human-subjects-research.

<Answer> The study protocol was approved by the ethics committee of Osaka University Hospital, the ethics committee of National Hospital Organization Osaka National Hospital, the ethics committee of Kinshukai Infusion Clinic, the ethics committee of Osaka Rosai Hospital, the ethics committee of NTT-West Osaka Hospital, the ethics committee of Sumitomo Hospital, the ethics committee of Toyonaka Municipal Hospital, the ethics committee of Otemae Hospital, the ethics committee of Sakai City Medical Center, the ethics committee of Itami City Hospital, the ethics committee of Nishinomiya Municipal Central Hospital, the ethics committee of Hyogo Prefectural Nishinomiya Hospital, the ethics committee of Osaka Police Hospital, the ethics committee of Yao Municipal Hospital, the ethics committee of National Hospital Organization Osaka-minami National Hospital, and the ethics committee of Higashiosaka City Medical Center. We described this on **lines 114-125** of the revised manuscript and added the same text to the "Ethics Statement" field of the submission form.

7. Please include captions for your Supporting Information files at the end of your manuscript, and update any in-text citations to match accordingly. Please see our Supporting Information guidelines for more information: http://journals.plos.org/plosone/s/supporting-information.

<Answer> We included captions for Supporting Information files at the end of the manuscript, and updated in-text citations.
