# Supplementary material for: Psychologic stress and disease activity in patients with inflammatory bowel disease: A multicenter cross-sectional study
Source: PLoS One. 2020 May 26;15(5):e0233365. doi: 10.1371/journal.pone.0233365 (PMC7250441; doi:10.1371/journal.pone.0233365)
Supplement: Attachment — Submitted filename: Araki Stress Response to Reviewers R2 v3.2.pdf [file pone.0233365.s007.pdf]

Response to Reviewer #1:

Thank you very much for reviewing our manuscript. We have now revised the manuscript in response to all comments received.

1. Please report the method to estimate P values for interactions in the Method section.

<Answer> To estimate P values for interactions, two-way analysis of variance was used for continuous variables and Cox regression analysis for categorical data. We have added the information in the Method section.

2. Please explain the possible biological mechanism which causes effect modification (i.e. interaction).

<Answer> In the current epidemiological study design, we cannot elucidate the definite biological mechanism underlying the effect modification. Multivariate analysis showed that patients with the PSTE group are predominantly female and do not think that other environmental factors exacerbated disease (Table 2). As described in the Discussion, psychologic stress can influence gut inflammation through various mechanisms via the hypothalamus–pituitary–adrenal axis and the autonomic nervous system, resulting in the production of proinflammatory cytokines, activation of macrophages, and alteration of intestinal permeability and gut microbiota [26-28]. Previous reports also show that female sex is associated with anxiety in IBD patients [30]. Increased psychologic stress in the PSTE group may augment the fear for exacerbation and thereby affect these mechanisms, whereas other factor(s) may affect disease activity in the non-PSTE group. We are currently conducting a basic research to explore the pathophysiological role of psychologic stress for intestinal inflammation. We have added the discussion in the Discussion of the revised manuscript.

3. The authors answered that there is no defined MCID for CES-D. Please mention the search method for this (i.e. database for searching and search terms). I briefly searched and found one article related to MCID (PMID: 27300327). There should be more articles related to this issue.

<Answer> In the paper presented by the Reviewer, the MCID for 15-item form of CES-D scale was investigated for German patients with depression, which is different from the 11-item CES-D we have used in the present manuscript. We searched papers using PubMed/MEDLINE with the search query of “11-item CES-D”[All Fields] AND “MCID”[All Fields] and no paper was found. We have additionally tried to search papers with the query of "MCID"[All Fields] AND "CES-D"[All Fields], three papers were found. MCID shown in the first paper was what the Reviewer #1 presented, the second was what the authors empirically considered for 20-item CES-D (PMID: 31037211), and third was not for CES-D (PMID: 31562683), so none of them can account for our query. Moreover, previous reports using CES-D score were targeting patients who had already been diagnosed as depression, which is totally different from our target of patients who are not diagnosed as depression in almost all cases. In the present study we would just like to investigate the statistically significant difference in the CES-D scores between the PSTE group and the non-PSTE group, and would not like to observe the decrease in the CES-D score by clinical intervention.

4. In the results section of the abstract, please report the details of the results such as proportion, point estimate, and 95% CI.

<Answer> We have added the details of the results within the word count limit in the revised abstract.

5. Figure 1 is difficult to follow. Moreover, the numbers written in the Patients section seem different from those in Figure 1. Please check them. If the numbers are correct, please clearly report the study flow. The authors should also report how many patients visited the study sites during the study period and how many were actually recruited.

<Answer> In the first draft, we displayed the depressive state and insomnia analysis charts separately. In the second submission we revised as the Reviewer #1 instructed us to combine them, which seemed to be harder to follow the study flow. We have now renewed **Figure 1** and clarified the study flow in the manuscript. And we actually recruited a total of 1078 cases, and the number of the patients who visited the study sites is unknown in this study design.

6. In the statistical analysis section, the description is too simple. Please report the details such as how they selected covariates for the logistic regression model. If the authors want to conduct causal inference, they need to select covariates based on theories instead of statistical covariate selection.

<Answer> In logistic regression analysis, the covariates with significant difference in the univariate analysis were selected. We have added the explanation to the Statistical analysis section.

7. In figure 3, the authors compared between remission and active patients with stratification of PSTE. To harmonize the way of showing results to figure 3, figure 4 should also show the comparison between

remission and active patients with stratification of PSTE without subgrouping CD/UC.

<Answer> In Figure 3 the factors related to PSTE are analyzed, while in Figure 4 the factors related to insomnia are analyzed, both of which are classified into the PSTE group and the non-PSTE group. Unlike CES-D, the insomnia score is a binary value, and if CD and UC are to be analyzed without distinction, disease activity must be expressed as a binary value in the active phase and the remission phase because the disease activity scores are different between CD and UC. Therefore, we have amended **Figure 4** as the active patient group and the remission patient group were set on the horizontal axis as in Figure 3, and the vertical axis was defined as the proportion of insomnia patients. Analysis by Pearson's chi-square test showed that, in the PSTE group, the proportion of insomnia patients was significantly higher in active patients than in those in remission, but the difference was not observed in the non-PSTE group. The P value of the interaction was 0.437, and no interaction was observed in both groups. These results are same as the former analysis, but thanks to the Reviewer's suggestion, we have now clearly showed that insomnia is associated with disease activity especially in patients with the PSTE group.

8. The authors should rethink the reasons to conduct multivariable analyses. They excluded "problems with work or family" from covariates because they thought that this was a potential confounder. If this is a confounder, it is better to adjust it to evaluate the independent association. However, the authors excluded it. Please explain for what purpose they conducted multivariable analyses.

<Answer> In the first analysis, the alternative "problems with work or family" was excluded from the analysis because it seemed to be strongly related to psychologic stress and the results might be confusing to the readers. When we actually put it in variables, problems with work and family was shown to be an

independent factor positively associated with PSTE, and other variables with significant difference in univariate analysis were remained as independent factors. These results indicate that patients in the PSTE group think that problems with work or family exacerbate the disease but other environmental factors do not. We have now amended **Table 2**.

9. In Table 2 and Table 3, it is difficult to understand the results. Please use same method to conduct univariate and multivariable analyses such as logistic regression models. Moreover, the method of covariate selection and purpose of these analyses are unclear.

<Answer> Table 2 analyzes factors related to PSTE, and Table 3 analyzes factors related to insomnia. In the first draft of Table 2 and 3, the results of the univariate analysis for all the variables were shown, but we deleted the variables without significant difference in the univariate analysis according to the Reviewer #1's suggestion. The analysis methods are the same. However, with the change in Figure 4 for the Question No.7, we would like to emphasize psychologic stress rather than insomnia, and have now deleted the Table 3 to avoid confusing.
